# *BMPR2* mutations and survival in pulmonary arterial hypertension: an individual participant data meta-analysis

**DOI:** 10.1016/S2213-2600(15)00544-5

**Published:** 2016-02

**Authors:** Jonathan D W Evans, Barbara Girerd, David Montani, Xiao-Jian Wang, Nazzareno Galiè, Eric D Austin, Greg Elliott, Koichiro Asano, Ekkehard Grünig, Yi Yan, Zhi-Cheng Jing, Alessandra Manes, Massimiliano Palazzini, Lisa A Wheeler, Ikue Nakayama, Toru Satoh, Christina Eichstaedt, Katrin Hinderhofer, Matthias Wolf, Erika B Rosenzweig, Wendy K Chung, Florent Soubrier, Gérald Simonneau, Olivier Sitbon, Stefan Gräf, Stephen Kaptoge, Emanuele Di Angelantonio, Marc Humbert, Nicholas W Morrell

**Affiliations:** a Department of Medicine, University of Cambridge School of Clinical Medicine, Cambridge, UK; b Department of Haematology, University of Cambridge School of Clinical Medicine, Cambridge, UK; c Department of Cardiology, Papworth Hospital, Cambridge, UK; d Université Paris–Sud, Faculté de Médecine, Université Paris-Saclay, Le Kremlin Bicêtre, France; e APHP, Centre de Référence de l'Hypertension Pulmonaire Sévère, Département Hospitalo-Universitaire Thorax Innovation, Service de Pneumologie, Hôpital de Bicêtre, Le Kremlin Bicêtre, France; f INSERM UMR_S 999, Laboratoire d'Excellence en Recherche sur le Médicament et l'Innovation Thérapeutique, Centre Chirurgical Marie Lannelongue, Le Plessis Robinson, France; g Thrombosis and Vascular Medicine Center, State Key Laboratory of Cardiovascular Disease, FuWai Hospital, Chinese Academy of Medical Sciences and Peking Union Medical College, Beijing, China; h Department of Experimental, Diagnostic and Specialty Medicine-DIMES, University of Bologna, Bologna, Italy; i Institute of Cardiology, University of Bologna, Bologna, Italy; j Department of Paediatrics, Vanderbilt University School of Medicine, Nashville, TN, USA; k Department of Medicine, Vanderbilt University School of Medicine, Nashville, TN, USA; l Department of Medicine, Intermountain Medical Center and the University of Utah School of Medicine, Salt Lake City, UT, USA; m Division of Pulmonary Medicine, Tokai University School of Medicine, Isehara, Japan; n Centre for Pulmonary Hypertension, Thorax Clinic, University Hospital Heidelberg, Heidelberg, Germany; o Institute of Human Genetics, University Hospital Heidelberg, Heidelberg, Germany; p Department of Cardio-Pulmonary Circulation, Shanghai Pulmonary Hospital, School of Medicine, Tongji University, Shanghai, China; q Division of Cardiology, Department of Medicine, Kyorin University School of Medicine, Tokyo, Japan; r Department of Pediatric Cardiology, Columbia University Medical Center, New York, NY USA; s Departments of Pediatrics and Medicine, Columbia University Medical Center, New York, NY USA; t Université Pierre et Marie Curie-Paris 6, Laboratoire d'Oncogénétique et Angiogénétique Moléculaire, Groupe Hospitalier Pitié-Salpétrière, Paris, France; u Department of Public Health and Primary Care, University of Cambridge, Cambridge, UK

## Abstract

**Background:**

Mutations in the gene encoding the bone morphogenetic protein receptor type II (*BMPR2*) are the commonest genetic cause of pulmonary arterial hypertension (PAH). However, the effect of *BMPR2* mutations on clinical phenotype and outcomes remains uncertain.

**Methods:**

We analysed individual participant data of 1550 patients with idiopathic, heritable, and anorexigen-associated PAH from eight cohorts that had been systematically tested for *BMPR2* mutations. The primary outcome was the composite of death or lung transplantation. All-cause mortality was the secondary outcome. Hazard ratios (HRs) for death or transplantation and all-cause mortality associated with the presence of *BMPR2* mutation were calculated using Cox proportional hazards models stratified by cohort.

**Findings:**

Overall, 448 (29%) of 1550 patients had a BMPR2 mutation. Mutation carriers were younger at diagnosis (mean age 35·4 [SD 14·8] vs 42·0 [17·8] years), had a higher mean pulmonary artery pressure (60·5 [13·8] vs 56·4 [15·3] mm Hg) and pulmonary vascular resistance (16·6 [8·3] vs 12·9 [8·3] Wood units), and lower cardiac index (2·11 [0·69] vs 2·51 [0·92] L/min per m^2^; all p<0·0001). Patients with *BMPR2* mutations were less likely to respond to acute vasodilator testing (3% [10 of 380] vs 16% [147 of 907]; p<0·0001). Among the 1164 individuals with available survival data, age-adjusted and sex-adjusted HRs comparing *BMPR2* mutation carriers with non-carriers were 1·42 (95% CI 1·15–1·75; p=0·0011) for the composite of death or lung transplantation and 1·27 (1·00–1·60; p=0·046) for all-cause mortality. These HRs were attenuated after adjustment for potential mediators including pulmonary vascular resistance, cardiac index, and vasoreactivity. HRs for death or transplantation and all-cause mortality associated with *BMPR2* mutation were similar in men and women, but higher in patients with a younger age at diagnosis (p=0·0030 for death or transplantation, p=0·011 for all-cause mortality).

**Interpretation:**

Patients with PAH and *BMPR2* mutations present at a younger age with more severe disease, and are at increased risk of death, and death or transplantation, compared with those without *BMPR2* mutations.

**Funding:**

Cambridge NIHR Biomedical Research Centre, Medical Research Council, British Heart Foundation, Assistance Publique-Hôpitaux de Paris, INSERM, Université Paris-Sud, Intermountain Research and Medical Foundation, Vanderbilt University, National Center for Advancing Translational Sciences, National Institutes of Health, National Natural Science Foundation of China, and Beijing Natural Science Foundation.

## Introduction

Pulmonary arterial hypertension (PAH) is a rare disorder characterised by progressive remodelling of the small pulmonary arteries resulting in increased pulmonary vascular resistance and ultimately right ventricular failure and death.^1^, ^2^ The diagnosis of PAH requires a mean pulmonary artery pressure of 25 mm Hg or more with a pulmonary artery wedge pressure of 15 mm Hg or less at right heart catheterisation in the absence of chronic thromboembolic, left heart, or respiratory disease.^3^ The classification of PAH includes idiopathic and heritable forms. Additionally, PAH might occur after drug or toxin exposure (eg, anorexigens) or in association with other conditions such as congenital heart disease, connective tissue disease, liver disease, or HIV infection.^4^

In 2000, heterozygous germline mutations in the gene encoding the bone morphogenetic protein receptor type II (*BMPR2*) were identified as the main genetic cause of familial PAH.^5^, ^6^ Over 300 different *BMPR2* mutations have been identified with a prevalence of greater than 75% in families with PAH.^7^, ^8^ BMPR-II is a receptor for the bone morphogenetic proteins (members of the transforming growth factor-β superfamily). Mutations in the *BMPR2* gene cause loss-of-function and reduced signalling downstream of the receptor. Subsequently, *BMPR2* mutations have been identified in apparently sporadic cases of idiopathic PAH with a frequency ranging from 11%^9^ to 40%.^10^ The occurrence of *BMPR2* mutations in sporadic PAH cases in the absence of a family history can be accounted for by the relatively low penetrance of *BMPR2* mutations (20–30%) and the occurrence of de novo mutations.^11^

**Research in context**

**Evidence before this study**

In the year 2000, mutations in the *BMPR2* gene were identified as the major genetic cause of pulmonary arterial hypertension (PAH). Some small studies have examined the effect of *BMPR2* mutations on the presentation, haemodynamic profile, and outcomes in patients with PAH. These studies suggested that those with *BMPR2* mutations present at a younger age with more severe derangements of cardiopulmonary haemodynamics. Due to a lack of statistical power, lack of adjustment for important factors such as age and sex, and confounding from inclusion of prevalent cases without necessary adjustments in many of these studies, the effect of *BMPR2* mutations on long-term outcomes has not been reliably established.

**Added value of this study**

By harmonising individual participant data from 1550 patients in eight published and unpublished studies, with updated follow-up, this study provides the most definitive assessment of the effect of *BMPR2* mutations on the haemodynamic profile at diagnosis and long-term outcomes in patients with PAH. This study has shown that possession of a *BMPR2* mutation is associated with an increased risk of death or transplantation and all-cause mortality. This association appears to be mediated by a more severe haemodynamic profile measured at diagnosis with the greatest proportion of the risk accounted for by the lower cardiac index in *BMPR2* mutation carriers. There was a strong interaction between the effect of a *BMPR2* mutation and age at diagnosis, such that the increased risk of death or transplantation and all-cause mortality associated with possession of a *BMPR2* mutation was greater in younger patients.

**Implications of all the available evidence**

Patients with PAH with underlying *BMPR2* mutations are younger at diagnosis, have more severe disease, and have a worse prognosis than patients without *BMPR2* mutations. The role of routine genetic testing for *BMPR2* mutations on the management of patients with PAH deserves further study.

Recent European guidelines for the management of PAH recommend offering genetic counselling and screening for *BMPR2* mutations to patients diagnosed with idiopathic, heritable, and anorexigen-associated PAH, mainly to enable predictive genetic testing of relatives.^12^ Studies have suggested that patients with PAH carrying causal *BMPR2* mutations present at an earlier age with more severe haemodynamic compromise.^13^, ^14^, ^15^, ^16^, ^17^ Although this might be expected to confer a worse survival, robust evidence describing the effect of *BMPR2* mutations on long-term outcomes in these patients is lacking, primarily due to the limited power of individual studies and survival bias.^18^, ^19^

We established the *BMPR2* Studies Collaboration to investigate the effect of *BMPR2* mutations on clinical phenotypes and long-term outcomes in patients with PAH. This international consortium has allowed central collation and harmonisation of participant data on 1550 patients with PAH from eight cohorts based in six different countries.

## Methods

### Data sources

We sought individual participant data from studies identified through systematic searches of the published literature using MEDLINE and Embase (search terms “BMPR2” and “pulmonary hypertension”, up to March 18, 2014), searches of conference proceedings (restricted to the English language), and discussions with investigators. Cohort studies were eligible for inclusion if they met the following criteria: included patients with idiopathic PAH, heritable PAH, or anorexigen-associated PAH; sequenced patients for *BMPR2* mutations; recorded baseline information on demographic and haemodynamic data at diagnosis, and for analysis of survival, recorded information on outcomes (death or transplantation). Inclusion of patients recruited to cohorts and registries since publication of original manuscripts was provided where available, including updated survival data. Details of contributing cohorts are presented in the appendix. Data from each study were obtained using a standardised spreadsheet; raw data were examined, and inconsistencies or irregularities were clarified with the relevant investigators.

Ethical approval was obtained for each of the individual studies included in this analysis and all participants provided informed written consent. This study followed the Preferred Reporting Items for Systematic Reviews and Meta-Analyses of Individual Participants Data (PRISMA-IPD) guidelines (checklist available in the appendix).^20^

### Study participants

All contributing studies used international criteria for the diagnosis of PAH.^21^ For the purpose of this study, the expert physician diagnosis of idiopathic PAH, heritable PAH, or anorexigen-associated PAH in a specialist centre was sufficient and data pertaining to the full range of investigations at diagnosis were not collected.

Vasodilator responsiveness was defined as a reduction in mean pulmonary arterial pressure of at least 10 mm Hg to a level below 40 mm Hg with no reduction in cardiac output after administration of inhaled nitric oxide, although some centres have historically used inhaled prostacyclin or intravenous prostacyclin, according to local practice. Treatment for PAH was prescribed consistent with prevailing international guidelines at the time of recruitment, at the discretion of the clinical team in each institution. Data regarding initial and subsequent PAH targeted therapy were not available for this analysis. Cohorts comprised a combination of incident patients, defined for the purposes of this study as those who were enrolled in their respective study and thus committed to genotyping within 6 months of PAH diagnosis, and prevalent patients who were enrolled more than 6 months after PAH diagnosis.

Patients were excluded from the analysis if they had PAH associated with conditions such as connective tissue disease, HIV, congenital heart disease, or portal hypertension. Furthermore, to avoid potential confounding from mutations in other genes or undetected *BMPR2* mutations, patients with a family history of PAH but with no identifiable *BMPR2* mutation were also excluded. Patients with a history of anorexigen exposure were included since *BMPR2* mutations have been recorded in these patients, and the disease is indistinguishable from idiopathic PAH.^4^, ^22^

### Outcomes

The primary outcome was the composite of death or lung transplantation. All-cause mortality was the secondary outcome. Patients contributed only the first outcome recorded during follow-up (ie, deaths preceded by transplantation were not included) because data regarding post-transplant survival were not available. Outcomes were censored if a patient was lost to follow-up or reached the end of the follow-up period. In analysis of all-cause mortality, patients were censored at the time of transplantation. Date of PAH diagnosis was defined as the date of diagnostic right heart catheterisation.

### Statistical analysis

Baseline characteristics of patients according to *BMPR2* mutation status were compared using *t* test for continuous variables and χ^2^ test for categorical variables. Associations of *BMPR2* mutation status with risk of death or transplantation and all-cause mortality recorded during follow-up were assessed using Cox proportional hazards regression models stratified by cohort and timing of study entry (ie, incident or prevalent). We used a one-stage stratified model rather than two-stage random effects model for our primary analysis because of the small number of participants in some studies. As a sensitivity analysis, we pooled data using a two-stage random effects model and assessed for heterogeneity between studies by calculating the *I*^2^ statistic and assessed statistical significance based on Cochran's Q test p value. Unless stated otherwise, hazard ratios (HRs) were adjusted for age at diagnosis and sex. The proportional hazards assumption was satisfied for the composite of death or transplantation and all-cause mortality. Survival curves comparing patients with and without *BMPR2* mutations were calculated using unadjusted Kaplan-Meier estimates and compared using the log-rank test.

To restrict the scope for potential bias due to inclusion of prevalent patients (ie, those diagnosed with PAH more than 6 months before study enrolment), Cox proportional hazards regression models and survival curves were fitted allowing for left truncation arising from the interval between diagnosis and enrolment. These patients were only included in the risk set from the time of study entry and were excluded if they entered the study more than 10 years after diagnosis. In patients for whom the date of enrolment in the study was not available, patients entered the risk set on the date they were genotyped for *BMPR2* mutations. Given that worse survival has been reported in incident patients compared with prevalent patients in observational studies,^18^ and a higher risk of PAH-related death or admission to hospital was reported in incident patients in a clinical trial population,^23^ Cox models were also stratified by timing of study entry. Data pertaining to familial clustering of individuals and mutations were not available; however, to account for this, survival models were adjusted for clustering by sets of individuals with the same mutation from the same cohort. Two studies were not included in the survival analysis (due to insufficient survival data) but were included in the description of demographic and haemodynamic characteristics.

We explored interactions between the effect of *BMPR2* mutation with age at diagnosis and sex within the one-stage stratified Cox models. The interaction with age at diagnosis was assessed with age as a continuous variable, with cases separated into three post-hoc groups according to age at diagnosis for illustrative purposes (<30 years, 30–50 years, and >50 years).

To assess the degree to which the association of *BMPR2* mutations with outcome was mediated by haemodynamic characteristics at diagnosis, we calculated the percentage of excess risk mediated (PERM) by three mediators thought likely to be in the causal pathway: pulmonary vascular resistance, cardiac index, and vasodilator responsiveness. Each of these mediators was added to the age-adjusted and sex-adjusted Cox proportional hazards models individually, and then all three simultaneously. The PERM, that is the degree to which the HR is attenuated by addition of the mediator in question, was calculated using the equation:

$$\left(\frac{{\text{HR}}_{\text{without}\;\text{mediator}}-{\text{HR}}_{\text{with}\;\text{mediator}}}{{\text{HR}}_{\text{without}\;\text{mediator}}-1}\right)\times 100$$

For this analysis, missing covariate data were imputed using multiple imputation by chained equations in those individuals included in the survival analysis, to generate ten datasets with complete covariates. Cox proportional hazards models were fitted within each imputed dataset and combined using Rubin's rules. This analysis was repeated using only cases that had complete data for all three mediators as a sensitivity analysis.

In an exploratory analysis, we compared haemodynamic parameters at presentation (compared using one-way ANOVA) and survival in patients by *BMPR2* mutation type. Mutations were assigned to one of five categories (frameshift, stop-gained, splice site or intronic, large deletions or exonal duplications, missense).

A two-sided p value less than 0·05 was considered statistically significant throughout. Statistical analyses were done using Stata (version 14; StataCorp, College Station, TX, USA).

### Role of the funding source

No funding bodies had any role in the design, conduct, analysis of this study, or writing of the manuscript. The corresponding author had full access to the data and had the final responsibility for the decision to submit this manuscript with the permission of all coauthors.

## Results

**Figure 1:**
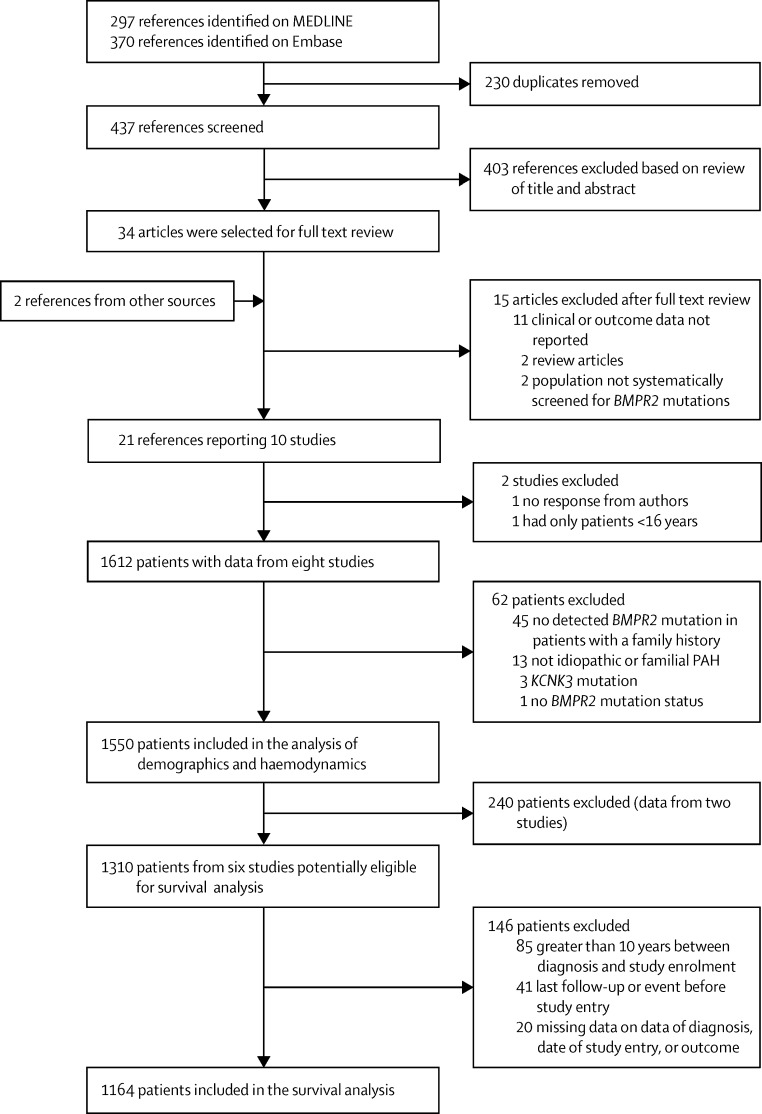
Study and patient selection

Figure 1 shows the inclusion and exclusion of studies and patients. Of ten studies identified, one eligible cohort (that involved 61 patients and was available only in abstract form)^24^ did not contribute data to the current analysis, and one cohort was excluded because it exclusively included 47 patients younger than 16 years.^25^ We analysed data from a total of 1550 patients with idiopathic, heritable, and anoroxigen-associated PAH from eight cohorts (appendix).^13^, ^14^, ^15^, ^16^, ^17^, ^26^, ^27^, ^28^ The mean age at diagnosis was 40·1 (SD 17·2) years, 72% (1105/1545 [data for five patients were unavailable]) were women, 60% (931/1550) were from western Europe, 18% (276/1550) from North America, and 22% (343/1550) from east Asia. Overall, 448 (29%) of 1550 patients had an identified *BMPR2* mutation and 86 (6%) of 1550 patients had a recorded history of anorexigen exposure. Histograms of the dates during which patients included in the survival analyses were diagnosed and recruited into these studies are shown in the appendix.

The proportion of patients with *BMPR2* mutations varied between studies (appendix). In patients with no recorded family history of PAH, a *BMPR2* mutation was identified in 17% (200/1174), whereas in patients with a family history of PAH a mutation was identified in 82% (202/247). Patients with a *BMPR2* mutation were younger at diagnosis (mean age 35·4 years *vs* 42·0 years, p<0·0001) and the proportion of patients with a *BMPR2* mutation was greater in those diagnosed at a younger age (37% [162/434] in those aged <30 years, 33% [187/562] in those aged 30–50 years, and 17% [75/451] in those aged >50 years at diagnosis; p<0·0001). A comparison of haemodynamic and functional parameters measured at the time of diagnosis between carriers and non-carriers of a *BMPR2* mutation is shown in table 1. Those carrying a *BMPR2* mutation had a higher mean pulmonary artery pressure and pulmonary vascular resistance, and lower cardiac index. No difference was recorded in the severity of symptoms assessed by New York Heart Association functional class or exercise capacity assessed by 6 min walk distance. Patients with a *BMPR2* mutation were less likely to respond to acute vasodilator testing (table 1).

**Figure 2:**
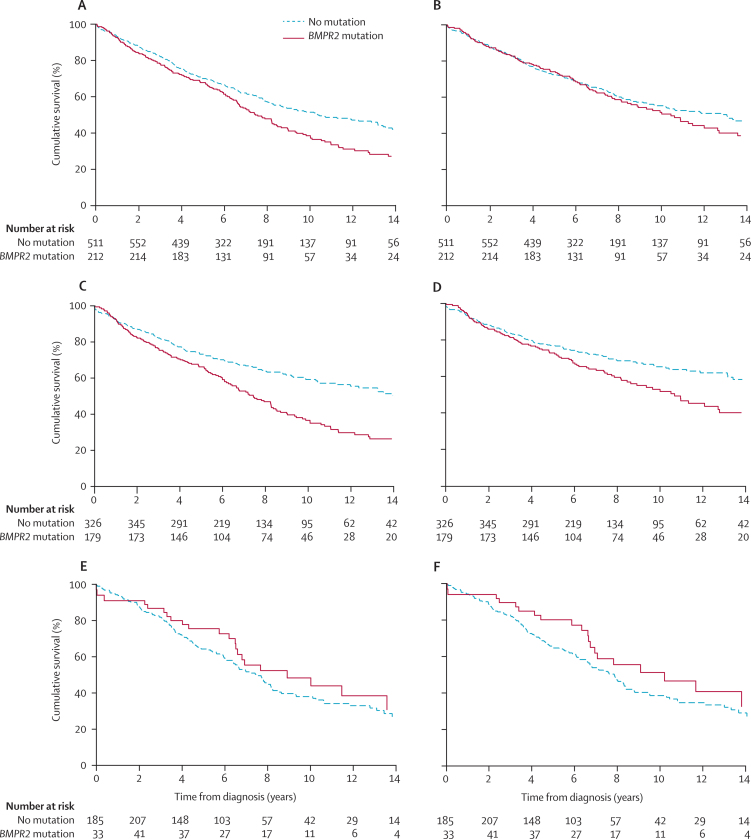
Kaplan-Meier survival curves according to *BMPR2* mutation status (A) Transplant-free survival, all patients (p=0·0016). (B) Overall survival, all patients (p=0·32). (C) Transplant-free survival, younger than 50 years at diagnosis (p<0·0001). (D) Overall survival, younger than 50 years at diagnosis (p=0·0032). (E) Transplant-free survival, older than 50 years at diagnosis (p=0·27). (F) Overall survival, 50 years or older at diagnosis (p=0·16). Survival curves are not adjusted for age at diagnosis or sex and are not stratified by study cohort.

Characteristics of the 1164 patients from the six studies that contributed to the survival analysis are shown in the appendix. Survival curves by *BMPR2* mutation status in the combined dataset are shown in figure 2. Of the 1164 patients, 723 (62%) were incident cases and 441 (38%) were prevalent cases. The median interval between diagnosis and study entry in the prevalent patients was 1·8 years (IQR 1·1–4·5). During 5870 person-years at risk (median duration of follow-up from diagnosis 5·4 years [IQR 3·0–8·2]), there were 354 deaths and 74 patients underwent lung transplantation. Age-adjusted and sex-adjusted HRs comparing *BMPR2* mutation carriers with non-carriers were 1·42 (95% CI 1·15–1·75; p=0·0011) for the composite of death or lung transplantation and 1·27 (1·00–1·60; p=0·046) for all-cause mortality (table 2). HRs were unchanged after adjusting for previous exposure to anorexigens. Addition of each of the three mediators assessed to the age-adjusted and sex-adjusted models attenuated the HRs associated with *BMPR2* mutation for both death or transplantation and all-cause mortality (table 2). Cardiac index mediated the greatest proportion of excess risk, accounting for 65% and 79% of the increased HR for death or transplantation and all-cause mortality, respectively. The PERM by the combination of pulmonary vascular resistance, cardiac index, and vasodilator responsiveness was 71% for death or transplantation and 100% for all-cause mortality. In the complete case sensitivity analysis (913 patients; appendix) the PERM by each mediator was similar.

**Figure 3:**
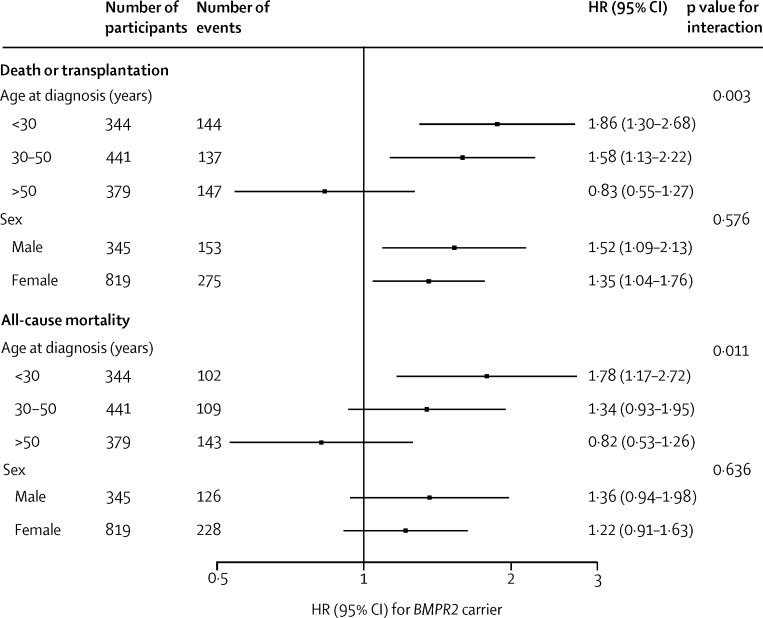
Hazard ratios (HRs) for the effect of a *BMPR2* mutation on death or transplantation and all-cause mortality by age at diagnosis and sex p value for interaction of *BMPR2* and age at diagnosis calculated with age at diagnosis as a continuous variable.

HRs associated with possession of a *BMPR2* mutation were similar in men and women (p=0·576 for death or transplantation, p=0·636 for all-cause mortality), but higher in patients with a younger age at diagnosis (p=0·0030 for death or transplantation, p=0·011 for all-cause mortality; figure 3, appendix). The interaction of *BMPR2* and age at diagnosis persisted after adjustment for potential mediators (appendix).

Similar results were recorded with meta-analysis using a two-stage approach with random effects (appendix). Between-study heterogeneity was modest, both for death or transplantation (*I*^2^=36·9% [95% CI 0–70]; p=0·16) and all-cause mortality (*I*^2^=20·1% [0–65]; p=0·28).

There were no significant differences in haemodynamic parameters at diagnosis between those with different mutation subtypes (appendix). Patients with missense mutations were slightly younger at diagnosis. No significant difference was recorded in the risk of death or transplantation or all-cause mortality among carriers of different types of *BMPR2* mutations (appendix).

## Discussion

To our knowledge, this meta-analysis of individual participant data from published and unpublished studies provides the most comprehensive standardised assessment of associations of *BMPR2* mutations with long-term outcomes in patients with idiopathic, heritable, and anorexigen-associated PAH.

We have shown that patients diagnosed with PAH have an increased risk of death or transplantation and all-cause mortality if they possess a mutation in the *BMPR2* gene. HRs associated with possession of a *BMPR2* mutation were similar in males and females, but greater with younger age at diagnosis. Furthermore, we have confirmed in this analysis the previously reported observations that patients with *BMPR2* mutations present at a younger age, have more severe haemodynamic compromise at diagnosis with higher mean pulmonary artery pressure and pulmonary vascular resistance and lower cardiac index, and are less likely to respond to acute vasodilator testing and more likely to undergo lung transplantation.^13^, ^14^, ^15^, ^16^, ^17^, ^26^

The precise mechanisms underlying the difference in survival in those with a *BMPR2* mutation remain unclear. We have found that after adjusting for pulmonary vascular resistance, cardiac index, and vasodilator responsiveness, HRs for death or lung transplantation and all-cause mortality were attenuated. The low number of *BMPR2* mutation carriers having vasodilator responsiveness, a phenotype associated with a good prognosis when treated with calcium-channel blocker therapy,^29^, ^30^ is consistent with a predominance of extensive vascular remodelling rather than vasoconstriction. Given that the greatest percentage of the excess risk associated with a *BMPR2* mutation was accounted for by reduced cardiac index at diagnosis, impaired right ventricular adaptation to increased afterload in those with *BMPR2* mutations could be an important factor, as has been suggested in preclinical studies.^31^

Our finding of a greater proportion of *BMPR2* mutations in younger age groups is consistent with the common observation that diseases with a major genetic contribution tend to present with an earlier onset. The strong interaction between *BMPR2* mutation status and age at diagnosis is of great interest and has not been reported before. Patients carrying a *BMPR2* mutation in which PAH manifests at a younger age might have a more severe mutation that not only results in more extensive pulmonary vascular remodelling or impaired right ventricular adaptation at diagnosis, but also results in more rapid progression of the disease process. This hypothesis is supported by the observation that the worse prognosis associated with a *BMPR2* mutation in patients diagnosed before the age of 30 years in this study was not completely attenuated after adjustment for pulmonary vascular resistance, cardiac index, and vasoreactivity.

Data from the UK PAH registry^32^ suggest that patients with PAH diagnosed after the age of 50 years are phenotypically distinct compared with younger patients. Older patients have a higher prevalence of cardiovascular comorbidities including systemic hypertension, atrial fibrillation, and diabetes. In the present study, although we did not collect data on comorbidities, we found that the proportion of patients with a *BMPR2* mutation is lower in those diagnosed after the age of 50 years than in younger patients. Additionally, we show that after adjusting for age and sex, mutations do not affect survival in these older patients, and prognosis might even be better in those with mutations. *BMPR2* mutations present in those who do not develop PAH until later in life might be less deleterious. Alternatively, in the older age group, comorbidities might outweigh the effect of *BMPR2* mutations on survival.

Our study confirms the relatively high frequency of *BMPR2* mutations in idiopathic and heritable PAH, and supports a central role for the *BMPR2* pathway in the initiation of this disease. Moreover, the effect of *BMPR2* mutation on survival suggests a role for BMPR-II dysfunction in the clinical progression of the disease. Both of these observations support further investigations into the potential targeting of the BMPR-II pathway for therapeutic intervention in PAH.^33^, ^34^

The main reason to test for the presence or absence of a *BMPR2* mutation in a patient with PAH is to guide predictive genetic testing in unaffected relatives. Although our findings show that *BMPR2* mutations are associated with a worse survival, the usefulness of this result for prognostic purposes might be restricted in the clinic, since the majority of this risk appears to be accounted for by the known haemodynamic predictors of mortality measured during the diagnostic assessment during right heart catheterisation. Despite this, in younger patients, in which the increased risk appears to persist after adjustment for these factors, albeit only in subgroup analyses, screening for mutations might add value, and this warrants further investigation.

Our analysis has major strengths. We had access to data for more than 95% of participants from eligible cohorts. We analysed individual participant data to avoid limitations of literature-based reviews. We had information on both all-cause mortality and death or transplantation. We studied clinically relevant subpopulations (such as by age and sex) reliably, exploiting the study's considerable statistical power. We avoided potential over-adjustment in the primary analysis by not adjusting for variables (eg, pulmonary vascular resistance, cardiac index, and vasoreactivity) that could mediate associations between *BMPR2* and death or transplantation and all-cause mortality. We ensured generalisability by studying cohorts located across east Asia, Europe, and North America.

Our analysis has some limitations. Studies included differed in their methods of recruitment and data collection, and in the proportion of familial cases and individuals with *BMPR2* mutations, which might explain the heterogeneity recorded between studies. Nevertheless, we obtained similar results to those in our primary analysis based on a stratified Cox proportional hazards model when we used a two-stage random effects meta-analysis model in sensitivity analysis. Additionally, given the evidence for interaction recorded between mutation status and age, the differences in age at diagnosis in different studies could partly explain the heterogeneity recorded in two-stage meta-analyses. The inclusion of prevalent patients in survival analyses can introduce bias; however, we addressed this in the Cox proportional hazards model by allowing for left truncation arising from the interval between diagnosis and study entry and also stratifying by timing of enrolment. Additionally, we observed no interaction between *BMPR2* mutation status and timing of enrolment. Finally, the lack of data regarding the timing and use of PAH-directed therapies might introduce some bias, although we believe any effect is likely to be very small. Indeed, if patients with *BMPR2* mutations were treated more aggressively due to their more severe haemodynamic derangements at diagnosis, this could have resulted in an attenuation of the association we have recorded.

By harnessing data from observational studies done worldwide, we have shown that in patients with idiopathic, familial, and anorexigen-associated PAH, the presence of a mutation in the *BMPR2* gene is associated with an increased risk of death or lung transplantation and all-cause mortality, particularly in those diagnosed at a younger age. Our analysis suggests that this association is largely mediated by the more severe haemodynamic derangements and low frequency of vasodilator responsiveness at diagnosis seen in those with *BMPR2* mutations.

## Figures and Tables

**Table 1:** Demographics and clinical measurements at diagnosis

		**All patients**	***BMPR2* mutation status**		
			Non-carriers (N=1102)	Carriers (N=448)	p value
Age at diagnosis (N=1447), years		40·1 (17·2)	42·0 (17·8)	35·4 (14·8)	<0·0001
Male sex		440/1545 (28%)	302/1097 (28%)	138/448 (31%)	0·20
Family history of PAH		202/1376 (15%)	..	202/402 (50%)	..
Body-mass index (N=1206), kg/m^2^		24·9 (9·1)	24·9 (10·6)	24·9 (5·9)	0·99
6-min walk distance (N=1072), m		378 (124)	374 (128)	388 (113)	0·088
NYHA functional class					0·38
	I–II	423/1426 (30%)	313/1031 (30%)	110/394 (28%)	
	III	896/1426 (63%)	647/1031 (63%)	249/394 (63%)	
	IV	107/1426 (8%)	72/1031 (7%)	35/394 (9%)	
Mean pulmonary artery pressure (N=1503), mm Hg		57·6 (15·0)	56·4 (15·3)	60·5 (13·8)	<0·0001
Pulmonary vascular resistance (N=1300), Wood units		14·0 (8·4)	12·9 (8·3)	16·6 (8·3)	<0·0001
Right atrial pressure (N=1253), mm Hg		8·2 (5·5)	8·0 (5·7)	8·6 (5·2)	0·065
Cardiac output (N=1202), L/min		3·98 (1·44)	4·20 (1·50)	3·50 (1·17)	<0·0001
Cardiac index (N=1358), L/min per m^2^		2·40 (0·88)	2·51 (0·92)	2·11 (0·69)	<0·0001
Vasodilator responder		157/1287 (12%)	147/907 (16%)	10/380 (3%)	<0·0001

Data are n/N (%) or mean (SD), unless otherwise stated. PAH=pulmonary arterial hypertension. NYHA=New York Heart Association.

**Table 2:** Proportion of excess risk mediated by haemodynamic variables at diagnosis

		**HR (95% CI) for *BMPR2* mutation**	**p value**	**PERM**
**Death or transplantation**				
Pulmonary vascular resistance				
	Adjusted for age and sex	1·42 (1·15–1·75)	0·0011	
	Adjusted for age, sex, and pulmonary vascular resistance	1·28 (1·03–1·58)	0·024	34%
Cardiac index				
	Adjusted for age and sex	1·42 (1·15–1·75)	0·0011	
	Adjusted for age, sex, and cardiac index	1·18 (0·95–1·47)	0·14	65%
Vasoreactivity				
	Adjusted for age and sex	1·42 (1·15–1·75)	0·0011	
	Adjusted for age, sex, and vasoreactivity	1·26 (1·02–1·57)	0·036	37%
Combined model				
	Adjusted for age and sex	1·42 (1·15–1·75)	0·0011	
	Adjusted for age, sex, pulmonary vascular resistance, cardiac index, and vasoreactivity	1·12 (0·89–1·41)	0·33	71%
**All-cause mortality**				
Pulmonary vascular resistance				
	Adjusted for age and sex	1·27 (1·00–1·60)	0·046	
	Adjusted for age, sex, and pulmonary vascular resistance	1·13 (0·89–1·43)	0·33	53%
Cardiac index				
	Adjusted for age and sex	1·27 (1·00–1·60)	0·046	
	Adjusted for age, sex, and cardiac index	1·06 (0·83–1·35)	0·67	79%
Vasoreactivity				
	Adjusted for age and sex	1·27 (1·00–1·60)	0·046	
	Adjusted for age, sex, and vasoreactivity	1·14 (0·89–1·45)	0·29	49%
Combined model				
	Adjusted for age and sex	1·27 (1·00–1·60)	0·046	
	Adjusted for age, sex, pulmonary vascular resistance, cardiac index, and vasoreactivity	1·00 (0·77–1·29)	0·98	100%

Hazard ratios (HRs) associated with possession of a *BMPR2* mutation after addition of each mediator individually to age-adjusted and sex-adjusted Cox proportional hazards models with the percentage of excess risk mediated (PERM) by each mediator. Missing data for mediators generated by multiple imputation.
