# Supplementary material for: BMPR2 mutations and survival in pulmonary arterial hypertension: an individual participant data meta-analysis
Source: Lancet Respir Med. 2016 Feb;4(2):129–37. doi: 10.1016/S2213-2600(15)00544-5 (PMC4737700; doi:10.1016/S2213-2600(15)00544-5)
Supplement: Supplementary appendix [file mmc1.pdf]

THE LANCET
Respiratory Medicine

## Supplementary appendix

This appendix formed part of the original submission and has been peer reviewed. We post it as supplied by the authors.

Supplement to: Evans JDW, Girerd B, Montani D, et al. *BMPR2* mutations and survival in pulmonary arterial hypertension: an individual participant data meta-analysis. *Lancet Respir Med* 2016; published online Jan 18. http://dx.doi.org/10.1016/S2213-2600(15)00544-5.
